# Geografische Ansätze in der Gesundheitsberichterstattung

**DOI:** 10.1007/s00103-020-03208-6

**Published:** 2020-08-28

**Authors:** Daniela Koller, Doris Wohlrab, Georg Sedlmeir, Jobst Augustin

**Affiliations:** 1 Institut für Medizinische Informationsverarbeitung, Biometrie und Epidemiologie – IBE, LMU München, München, Deutschland; 2 Referat für Gesundheit und Umwelt, Landeshauptstadt München, Bayerstr. 28a, 80335 München, Deutschland; 3 Institut für Versorgungsforschung in der Dermatologie und bei Pflegeberufen (IVDP), FG Gesundheitsgeografie, Universitätsklinikum Hamburg-Eppendorf, Hamburg, Deutschland

**Keywords:** Gesundheitsberichterstattung, Geografische Methoden, GIS, Regionale Variation, Gesundheitsatlas, Health monitoring, Geographic methods, GIS, Regional variation, Gesundheitsatlas

## Abstract

Das Interesse an geografischen Darstellungen in der Gesundheitsberichterstattung (GBE) ist in den letzten beiden Jahrzehnten stark gewachsen. Gesundheitsdaten können mit diesen Methoden anschaulich und zielgruppenorientiert visualisiert werden. Neue technische Möglichkeiten und die breitere Verfügbarkeit von Daten tragen zur verstärkten Anwendung in der GBE bei. In diesem Artikel soll gezeigt werden, welche geografischen Ansätze in der GBE auf Bundes‑, Länder- und Kommunalebene jeweils aktuell verfolgt werden. Insbesondere soll dabei auf die verwendeten Methoden fokussiert werden.

Es wird gezeigt, dass auf Bundesebene geografische Methoden z. B. in der Surveillance angewendet werden; auf Länderebene gibt es z. B. Gesundheitsatlanten und auf der Kommunalebene verschiedene geografische Analysen. Die methodische Spannweite reicht von einfacheren Kartendarstellungen auf unterschiedlichen Aggregationsebenen bis hin zu komplexeren Verfahren wie raum-zeitlichen Darstellungen und räumlichen Glättungsverfahren.

Fehlender Datenzugang oder datenschutzrechtliche Aspekte behindern noch häufig die Verbindung mit weiteren Datenquellen oder kleinräumigere Darstellungen. Vor allem ein besserer Zugang zu Daten auf kleinräumiger Ebene könnte die GBE aber erheblich erleichtern. Die Bevölkerung und Entscheidungsträger könnten dadurch noch umfassender informiert und folglich die Gesundheit und die gesundheitliche Versorgung der Bevölkerung verbessert werden.

## Abstract (en)

The interest in using geographic methods for health monitoring has grown strongly over the last two decades. Through these methods, analysis and visualization of health data can be more focused and target-group specific. The application in health monitoring is possible mostly due to broader technical possibilities and more available datasets. In this article, we show which geographic aspects are adapted in health monitoring at different levels (federal, state, municipality).

For example, at the federal level, surveillance methods are used; at the state level health atlases are created; and on the municipality level geographic analyses are performed for possible public health interventions.

Methods range from simple maps on different levels of aggregation to more complex methods like space-temporal visualization or spatial-smoothing methods. While the technical possibilities are in place, a broader implementation of geographic methods is mostly hindered by missing data access to small-area information and data protection policies. Better access to data could especially improve the possibility for geographic methods in health monitoring and could inform the population and decision makers to inform and improve population health or healthcare.

## Einleitung

Bereits im Jahr 2000 erschien ein Artikel zur regionalen und kommunalen Gesundheitsberichterstattung (GBE), in dem die Autoren Jacob und Michels zu einer besseren Datenbasis für regionale GBE aufriefen und die Relevanz der Identifizierung regionaler Ungleichheiten unterstrichen [1]. Seitdem sind 20 Jahre vergangen, in denen sich nicht nur die Datenlage, sondern auch die Softwaremöglichkeiten stark verbessert haben.

Die verschiedenen Aspekte der GBE werden in dem vorliegenden Themenheft dargestellt und diskutiert. Die GBE ist sehr breit aufgestellt, sowohl in Bezug auf die thematische Bandbreite wie auch auf die Zielgruppen. Sie informiert mithilfe von Berichten und Informationssystemen über Gesundheitszustand, Gesundheitsversorgung oder Gesundheitsdeterminanten der Bevölkerung und soll gesundheitsrelevante Programme unterstützen [2]. Dieser Beitrag soll zeigen, wie eine räumliche Betrachtung die Ziele der GBE („Daten für Taten“) unterstützen kann.

Geografische, das heißt raumbezogene Analysen sind mittlerweile ein fester Bestandteil von Public Health, Epidemiologie, Versorgungsforschung und GBE. Diese Entwicklung ist vor allem darauf zurückzuführen, dass in den letzten Jahren der Zugang zu Gesundheitsdaten mit regionalem Bezug einfacher wurde und dass es inzwischen diverse anwenderfreundliche Softwarelösungen gibt, mittels derer geografische Analysen durchgeführt und deren Ergebnisse in Form von Karten visualisiert werden können. Die Erforschung medizin- bzw. gesundheitsgeografischer Themen blickt auf eine viel längere Geschichte zurück.

Im deutschsprachigen Raum wurde der Begriff der *medizinischen Geografie* vor allem von Leonhard Ludwig Finke geprägt: Der deutsche Arzt beschrieb Ende des 18. Jahrhunderts bereits die erste medizinische Landkarte; August Hirsch, ein deutscher Medizinhistoriker, publizierte ca. 70 Jahre später ein dreibändiges Handbuch zur historisch-geografischen Pathologie [3, 4]. In den vergangenen Jahrzehnten stand in der Forschungsrichtung vor allem der Einsatz von Geoinformationssystemen (GIS) im Vordergrund. Die so entstandenen Karten stellen einerseits „klassische“ krankheitsökologische Informationen dar (also die regionale Verbreitung einer Erkrankung auf Basis regionaler Einheiten, wie beispielsweise Bundesländer oder Kreise/kreisfreie Städte), analysieren weitergehend aber auch zeitliche Zusammenhänge zwischen Raum und Gesundheit, Zugangsmöglichkeiten zu Versorgungseinrichtungen oder die Präsenz von Kontaktnetzwerken, um nur einige Beispiele zu nennen [5–8].

Einhergehend mit dieser Entwicklung ist die stetige Zunahme von Gesundheitsatlanten. Dabei handelt es sich um eine Sammlung von Karten, in denen gesundheitsspezifische Sachverhalte mit räumlichem Bezug gebündelt dargestellt werden. Das vermutlich bekannteste Werk sind die Publikationen im Rahmen des Dartmouth Atlas of Health Care [9–11], in dem mittels Karten und begleitender Texte Gesundheitsdaten (z. B. zur Häufigkeit von Erkrankungen, zur Gesundheitsversorgung) für die Vereinigten Staaten von Amerika dargestellt werden.

Gesundheitsatlanten können primär nach dem Ausgabemedium unterschieden werden: Sie erscheinen online oder in gedruckter Form. Mittlerweile nimmt die Zahl der Onlineatlanten im Vergleich zu den gedruckten Atlanten deutlich zu. Der Vorteil der Onlineatlanten liegt vor allem in der einfacheren Aktualisierbarkeit. Neben dem Ausgabeformat lassen sich diese Werke auch darin unterscheiden, ob es sich eher um einen Atlas im eigentlichen Sinne handelt, das heißt, die Karte mit ihrer Aussage im Vordergrund steht, oder ob es sich eher um einen Bericht zu einem spezifischen Gesundheitsthema handelt, der mit Karten illustriert wird. Darüber hinaus kann auch nach Zielgruppe, Datenquellen, Ergebnisaufbereitung oder auch Themen (Epidemiologie, Prävention etc.) unterschieden werden.

In Deutschland gibt es rund 50 Werke [12], die als „Gesundheitsatlas“ bezeichnet werden können oder den Charakter eines Berichts mit Karten haben. Zu ersteren Werken gehören beispielsweise der Versorgungsaltas des Zentralinstituts für die kassenärztliche Versorgung (Zi) als webbasierte Plattform [13] oder der Krebsatlas Schleswig-Holstein [14] als gedrucktes Exemplar. Der BKK Gesundheitsatlas [15] oder das Gesundheitsmonitoring des Robert Koch-Instituts (RKI; [16]) haben beispielsweise eher den Charakter eines Berichtes, in dem zur Veranschaulichung Karten hinzugefügt wurden. Grundsätzlich ist zu erwähnen, dass sich die Werke hinsichtlich ihrer Qualität unterscheiden und dahin gehend, ob bei ihrer Erstellung etwa Leitlinien wie die „Gute Epidemiologische Praxis“ [17] oder die „Gute Kartographische Praxis im Gesundheitswesen“ [18] berücksichtigt werden.

Dieser Beitrag will einen Blick auf die aktuell vorhandenen Instrumente geben, die speziell von der GBE für regionale Analysen genutzt werden. In den folgenden 3 Abschnitten werden die 3 administrativen regionalen Ebenen der GBE, also die Bundes‑, Landes- und Kommunalebene betrachtet. Ein spezieller Fokus wird auf die angewandten Methoden der geografischen Visualisierung gelegt und diese an jeweils 2 Beispielen dargestellt.

## Geografische Methoden in der GBE auf Bundesebene

Auf Bundesebene findet eine umfangreiche GBE statt, die in unterschiedlicher Ausprägung geografische Analysen implementiert. Die umfassenden Gesundheitsberichte (zuletzt erschienen 2015) stellen vereinzelte Indikatoren regional dar, wobei der Fokus auf Ebene der Kreise und kreisfreien Städte oder Bundesländer liegt. Neben dieser Quelle bilden insbesondere die großen Gesundheitssurveys die Datengrundlage für Analysen. Hier sind vor allem die Studie „Gesundheit in Deutschland aktuell“ (GEDA), die „Studie zur Gesundheit Erwachsener in Deutschland“ (DEGS) und die „Studie zur Gesundheit von Kindern und Jugendlichen in Deutschland“ (KiGGS) zu nennen [19–21], wobei deren Anwendung bei kleinräumigen Analysen noch als schwierig einzuschätzen ist [22]. Einige regionale Analysen wurden auf Basis dieser Studien allerdings durchgeführt, teilweise auf höherer räumlicher Aggregation (also bspw. auf Bundeslandebene, nur im Ost-West-Vergleich, auf pseudonymisierter Kreisebene). Die Daten der GEDA-Studie lassen auch Analysen auf Kreisebene zu [23–27]. In einer Übersichtsarbeit von Thißen et al. wird dargestellt, dass vor allem amtliche Statistiken als Datenquellen für die geografischen Analysen im Gesundheitsmonitoring verwendet werden [28].

Im Folgenden sollen 2 Beispiele geografischer Methoden gezeigt werden: die Berichterstattung über den COVID-19-Ausbruch (welcher zur Zeit der Fertigstellung dieses Artikels aktuell ist) und die kontinuierlich durchgeführte Surveillance von akuten Atemwegsinfekten und Influenza.

### Berichterstattung zu COVID-19

**Figure 1:**
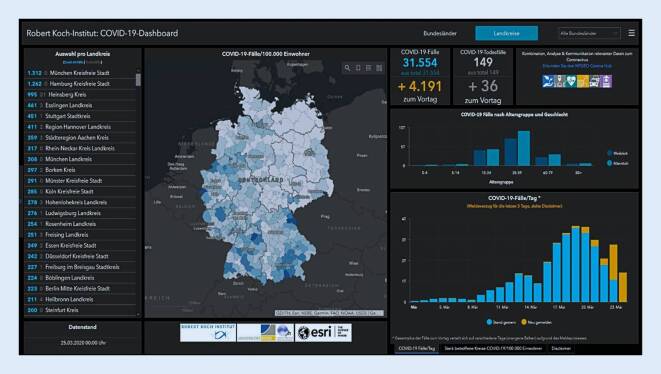

Das „COVID-19-Dashboard“ (Abb. 1) ist eine interaktive Onlinekarte, die im Jahr 2020 speziell für den pandemischen Ausbruch des Virus SARS-CoV‑2 in Kooperation des RKI mit dem GIS-Softwarehersteller ESRI und dem GeoHealth Centre Bonn entwickelt wurde. Ziel ist die kontinuierliche Darstellung der Anzahl von Erkrankungs‑, Todes- und Genesungsfällen zur Informierung der Bevölkerung. Die Daten basieren auf den gemeldeten Fällen der Landesämter, die an das RKI weitergegeben werden. Die Aktualisierung findet täglich statt, wobei ein Melde- und Übermittlungsverzug zu beachten ist. Die Daten werden in Form einer Karte auf Bundesland- oder Kreisebene dargestellt, begleitende Grafiken geben zusätzliche Informationen zur Fallzahlentwicklung. Die Anwender können zwischen Bundesländern und Kreisen/kreisfreien Städten wählen und bekommen so einen aktuellen Stand der Erkrankungsfälle, ein wichtiger Bestandteil der Aufklärung bei dieser Pandemie.

Ähnliche Darstellungsmethoden werden sowohl in einzelnen Kreisen und Städten für die eigene Region eingesetzt [29], aber auch in anderen Ländern, wie z. B. der Schweiz, Liechtenstein, Österreich und Slowenien. Die Weltgesundheitsorganisation (WHO) stellt ebenfalls ein Dashboard bereit, welches Informationen zu den europaweiten Erkrankungsfällen gibt.

### Surveillance von Atemwegsinfektionen und Influenza

Als zweites Beispiel soll die Surveillance dienen, welche auf Bundesebene unter dem Gesichtspunkt der geografischen Analysen besonders zu erwähnen ist. Ziel ist hier unter anderem die kontinuierliche Darstellung von Atemwegs- und speziell Grippeerkrankungen. Die Arbeitsgemeinschaft Influenza (AGI) des RKI berichtet während der Winter- und Frühjahrsmonate wöchentlich die Anzahl der diagnostizierten Atemwegserkrankungen und Influenzafälle sowie der Arztkontakte aufgrund akuter Atemwegserkrankungen [30].

**Figure 2:**
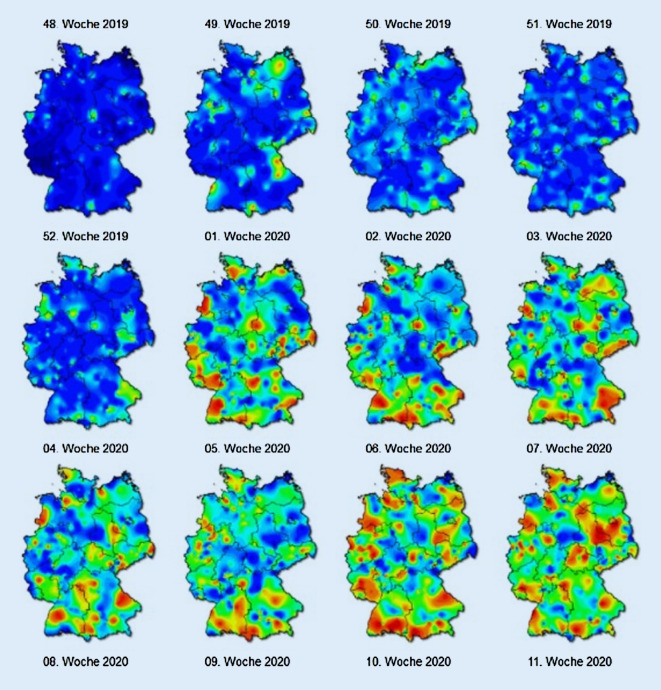

Die Daten werden über Sentinel-Praxen ermittelt, die über das gesamte Bundesgebiet verteilt sind (für weitere Hintergründe zu Erhebung und Methodik siehe [30]). Um erhöhte Fall bzw. Konsultationszahlen regional zu identifizieren, wird eine Abweichung vom Mittelwert über alle Praxen hinweg ermittelt. Die geografische Darstellung basiert auf einer Interpolation, basierend auf dem Kriging-Verfahren [30–32], sodass ein deutschlandweites Bild zu normalen bzw. erhöhten Aktivitäten bei akuten Atemwegserkrankungen entsteht. Neben der kartografischen Darstellung wird ein ausführlicher Influenzabericht erstellt, welcher während der Erkältungssaison im Winter ebenfalls wöchentlich aktualisiert wird und auf der Website der Arbeitsgemeinschaft Influenza zum Download zur Verfügung steht (Abb. 2).

## Geografische Methoden in der GBE auf Länderebene

Auf Ebene der Länder gibt es diverse Produkte der regionalen GBE, in denen Themen aus dem Länderindikatorensatz [33] aufgegriffen werden. Dieser Indikatorensatz umfasst ca. 300 Gesundheitsindikatoren und dient als Basis für die GBE auf Landesebene. Die Indikatoren werden unter anderem online in der Form von Atlanten publiziert. Tab. 1 zeigt eine Auswahl von Gesundheitsatlanten (nicht ausschließlich GBE-Atlanten). Diese sind ihren Inhalten nach entweder allgemeiner gehalten (z. B. Gesundheitsprofile Bayern) oder fokussieren auf spezifische Indikationen bzw. Indikationsgruppen, wie etwa die Krebsatlanten. Exemplarisch sollen an dieser Stelle der Gesundheitsatlas Baden-Württemberg sowie der Krebsatlas Schleswig-Holstein kurz vorgestellt werden.

**Table 1:** 

Bundesland	Name	Institution	Quelle
Bayern	Gesundheitsatlas Bayern	Bayerisches Landesamt für Gesundheit und Lebensmittelsicherheit	[34]
Bayern	Gesundheitsprofile Bayern	Bayerisches Landesamt für Gesundheit und Lebensmittelsicherheit	[53]
Baden-Württemberg	Gesundheitsatlas Baden-Württemberg	Landesgesundheitsamt	[52]
Nordrhein-Westfalen	Gesundheitsatlas Nordrhein-Westfalen	Landeszentrum Gesundheit	[54]
Rheinland-Pfalz	Gesundheitsatlas Rheinland-Pfalz	Statistisches Landesamt	[55]
Niedersachsen	Interaktiver Bericht des epidemiologischen Krebsregisters Niedersachsen	Epidemiologisches Krebsregister	[56]
Schleswig-Holstein	Krebsatlas Schleswig-Holstein	Institut für Krebsepidemiologie e. V.	[14]
Hamburg	Morbiditätsatlas	Behörde für Gesundheits- und Verbraucherschutz	[57]

### Gesundheitsatlas Baden-Württemberg und Krebsatlas Schleswig-Holstein

**Figure 3:**
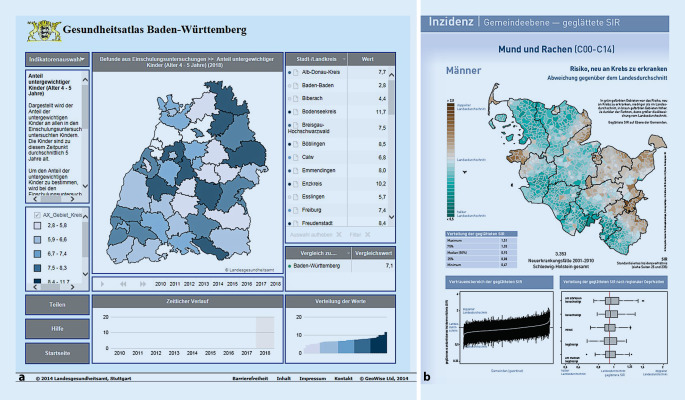

Der Gesundheitsatlas Baden-Württemberg (Abb. 3a) basiert auf einer Onlineplattform und greift die Themen Bevölkerung, medizinische Versorgung, Gesundheitszustand, Gesundheitsförderung, Prävention sowie Gesundheitsausgaben und Kosten auf. Die Daten werden je nach Datenquelle so aktuell wie möglich dargestellt und sind auch als Download verfügbar, sodass potenzielle Nutzer die Daten selbst verarbeiten und Karten produzieren können. Es wird die räumliche Ebene der Kreise und kreisfreien Städte Baden-Württembergs abgebildet. Eine Auswahl verschiedener Indikatoren kann zu den jeweiligen Themen aufgerufen werden. Die Indikatoren werden in einem kleinen Desktop-Fenster zusammen mit der Karte, einem Diagramm zum zeitlichen Verlauf des ausgewählten Indikators sowie einem Datenfenster angezeigt. Zu erwähnen ist, dass es sich bei dem Portal um einen interaktiven Atlas handelt. Der Nutzer kann zwar nicht etwa die Anzahl der Klassen, d. h. die Einteilung von Merkmalswerten, auswählen, sich jedoch mittels einer Mouse-over-Funktion beispielsweise den zeitlichen Verlauf eines Indikators für einzelne Kreise und kreisfreie Städte zeigen lassen. Der Atlas basiert auf der Software „InstantAtlas“, welche auch von anderen Landesbehörden für die GBE eingesetzt wird, wie beispielsweise für den *Gesundheitsatlas Bayern* [34] oder in Nordrhein-Westfalen. In diesen werden *Profile für Kreise und kreisfreie Städte *dargestellt, in denen Daten zu verschiedenen gesundheitsrelevanten Themen visualisiert werden [35].

Neben dem erwähnten Gesundheitsatlas Baden-Württemberg soll hier der Krebsatlas Schleswig-Holstein (Abb. 3b) Erwähnung finden. Krebsregister sind verpflichtend, die gesetzliche Regelung liegt bei den Ländern. Die meisten Krebsregister publizieren Ergebnisse nicht nur auf Landesebene, sondern auch auf Ebene der Kreise und kreisfreien Städte. Bundesweit werden die Daten sowohl im *GEKID-Atlas* als auch im Bericht *Krebs in Deutschland* veröffentlicht [36–38]. Ziele der Krebsregister sind es, die Früherkennung auszuweiten und zu einer höheren Versorgungsqualität beizutragen.

Der Krebsatlas Schleswig-Holstein ist keine interaktive, onlinebasierte Plattform. Er kann als PDF heruntergeladen werden und fokussiert sich inhaltlich auf die Häufigkeit von Krebserkrankungen in Schleswig-Holstein auf Ebene der Kreise und kreisfreien Städte und Gemeinden. 18 verschiedene Krebserkrankungen (z. B. Darm, Lunge, malignes Melanom der Haut) werden in Form von Einzelberichten hinsichtlich Inzidenz, Überleben und Mortalität mit statistisch geglätteten und ungeglätteten Daten als Abweichung vom Landesdurchschnitt in Form einer Karte dargestellt. Darüber hinaus werden Zusatzinformationen zu statischen Verteilungen, Zeitreihen oder Assoziationen zur regionalen Deprivation bereitgestellt. Die zugrunde liegenden Daten sind aus einer Tabelle ersichtlich. Bei diesem Atlas ist die ausführliche Beschreibung der Methodik hervorzuheben, die neben einer zur Verfügung stehenden Interpretationshilfe für ein hohes Maß an Transparenz sorgt.

## Geografische Methoden in der GBE auf Kommunalebene

Ziel kommunaler Planungen ist es, gleichwertige Lebensverhältnisse für die Bevölkerung einer Kommune zu schaffen, nicht zuletzt um Chancengerechtigkeit für verschiedene Bevölkerungsgruppen zu schaffen bzw. Benachteiligungen zu vermeiden oder auszugleichen und im Sinne der Daseinsvorsorge lebenswichtige Güter und Dienstleistungen bereitzustellen (im Sinne von § 2 Grundsätze der Raumordnung des Raumordnungsgesetzes (ROG) und Art. 28 Abs. 2 Grundgesetz für die Bundesrepublik Deutschland).

Um dies gewährleisten zu können, sind die Analyse und das Wissen um die sozialräumliche Verteilung gesundheitsbezogener Dienstleistungen, gesundheitsbezogener Indikatoren zum Gesundheitszustand/-verhalten, zu den Bedarfen, Ressourcen, Versorgungslücken sowie den Wünschen der Bürgerinnen und Bürger unabdingbare Voraussetzung. Jedoch ist gerade eine kleinräumige Darstellung innerhalb von Kommunen oft schwierig, da Daten auf Ebene der Stadtbezirke (oder noch kleinräumiger) nur selten erhoben werden bzw. nur eingeschränkt verfügbar gemacht werden. Kartografische Darstellungen auf Ebene der Kommune sind vor allem dann sinnvoll für Planungen – im Sinne von „Daten für Taten“ –, wenn gesundheits-, umwelt- und soziallagenbezogene Daten kleinräumig, d. h. unterhalb der gesamtstädtischen Ebene, zur Verfügung stehen. Für Daten zur sozialen Lage und für viele umweltbezogene Daten ist diese Voraussetzung erfüllt.

Es gibt eine Reihe Beispiele, wie auf kommunaler Ebene geografische Ansätze angewandt werden, um Gesundheitsthemen zu visualisieren (siehe beispielsweise Frankfurt am Main, Magdeburg oder Oberhausen [39–41]), wobei diese oft in Form von Sozialstrukturatlanten nicht nur spezifische Gesundheitsthemen abbilden. In diesem Absatz soll anhand zweier Beispiele aus der Landeshauptstadt München dargestellt werden, wie kartografische Darstellungen in der Gesundheits- bzw. der Umweltberichterstattung zu einem Mehr an gesundheitlicher Chancengleichheit beitragen können. Hierzu werden Analysen der Schuleingangsuntersuchung sowie die Berechnung eines *Walkability-Index* zur Messung der Fußgängerfreundlichkeit der Stadt München vorgestellt.

### Schuleingangsuntersuchung in München

In der Landeshauptstadt München werden trotz der vorhandenen Restriktionen, wie etwa schwieriger Datenverfügbarkeiten (s. Diskussion), geografische Themen in der GBE aufgegriffen und aufbereitet. Der Umgang mit mangelhafter Verfügbarkeit der Daten auf Adressebene wird exemplarisch an den Auswertungen der Schuleingangsuntersuchung aufgezeigt. Die der GBE vom Landesamt für Gesundheit und Lebensmittelsicherheit zur Verfügung stehenden Daten beinhalten die um die letzte Ziffer reduzierten 4‑stelligen Postleitzahlen. Hierfür wurden künstlich eigene Regionen kreiert – jenseits aller vorhandenen, städtischen administrativen Einteilungen, auf deren Ebene kleinräumigere Betrachtungen immerhin möglich sind, die aber in einigen Fällen eine Verschneidung mit anderen Daten überhaupt nicht zulassen und in anderen Fällen nur mit sehr hohem Aufwand.

Die Vorgehensweise wurde trotzdem gewählt, da die regionale Aufbereitung der Indikatoren der Schuleingangsuntersuchung zur frühen Erkennbarkeit von potenziellen Problemen und für die Planung von präventiven Maßnahmen, wie etwa im Projekt „München – gesund vor Ort“ (gefördert nach §20 SGB V) oder für die Bildungslokale, von hoher Bedeutung sind.

**Figure 4:**
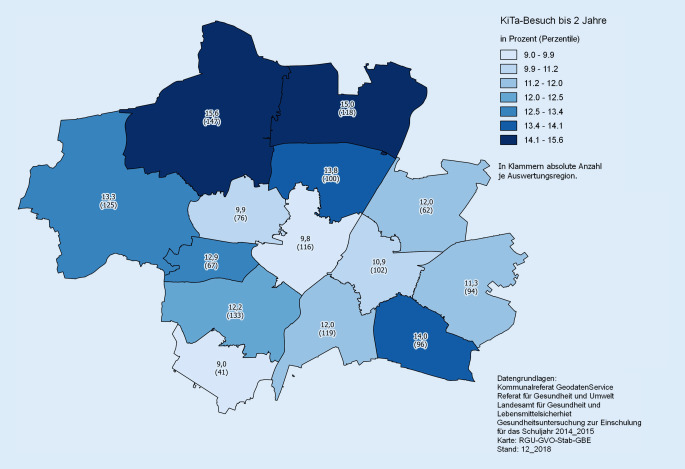

Abb. 4 zeigt die Besuchsdauer in Kindertageseinrichtungen bis maximal 2 Jahre und stammt aus der Analyse der Schuleingangsuntersuchung [42]. Die Karte zeigt klare regionale Trends innerhalb der Stadt, wonach in einigen Gebieten die Betreuung im Vorschulalter weniger häufig in Anspruch genommen wird als in anderen. Hier ist zukünftig ein Daten-Linkage zu beispielsweise Sozialdaten geplant, um Hintergründe und Auswirkungen analysieren zu können. Zudem werden Optionen ausgelotet, wie die Auswertungen auf kleinräumigerer Ebene, im Rahmen vorhandener administrativer Einteilungen, realisiert werden können.

### Walkability-Index für München

Als zweites Beispiel soll die Schaffung eines *Walkability-Index* vorgestellt werden. Dem Fußverkehr als gesundheitsförderliche Mobilitätsform wird im Vergleich zu anderen Mobilitätsformen von der Planung meist weniger Aufmerksamkeit gewidmet. Methodisch bestehen nach wie vor Defizite bei der Erfassung des Fußgängerverkehrs und des damit verbundenen Potenzials für die Entwicklung entsprechender Strategien der Umwelt- und Gesundheitsvorsorge. Das ursprünglich in den USA entwickelte und mittlerweile international adaptierte und weiterentwickelte Konzept der *Walkability* liefert ein Maß für die Möglichkeit, sich in der gegebenen physischen und soziokulturellen Umwelt zu Fuß zu bewegen, und steht damit auch in direktem Zusammenhang mit dem Begriff der Umweltgerechtigkeit. Im engeren Sinne beschreibt *Walkability* die Fußgängerfreundlichkeit der gebauten Umwelt aus verkehrsplanerischer Sicht unter Berücksichtigung der Flächennutzung und der Dichte des Wegenetzes. Dabei wird davon ausgegangen, dass eine fußgängerfreundlichere Umgebung gesundheitliche Vorteile für die Bevölkerung hat [43–46].

Für die Stadt München wurde ein Walkability-Index durch die Umweltberichterstattung des Referats für Gesundheit und Umwelt der Landeshauptstadt München im Projekt „Umweltgerechtigkeit in deutschen Städten“ erstellt. Dieses Projekt wurde von 2016 bis 2018 unter Leitung des Deutschen Instituts für Urbanistik (DIFU) und in Zusammenarbeit mit PLAN-HAI-21 sowie den Partnerstädten Kassel und Marburg durchgeführt.

Für den Index wurden Datensätze verschiedener Quellen zusammengeführt (u. a. Einwohnerzahlen, Flächennutzungsplan, Grünflächen) und über Geoinformationssysteme und OSMnx (ein Werkzeug, welches auf Basis der OpenStreetMap-Daten Straßennetzwerke darstellen kann) verarbeitet.

**Figure 5:**
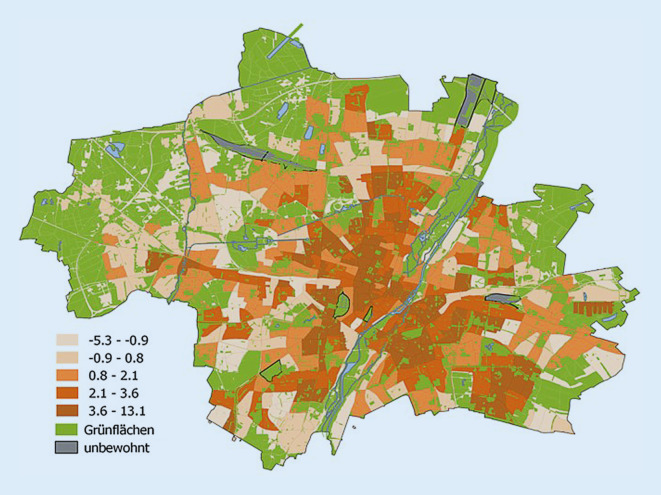

Der Ansatz basiert auf der grundlegenden Arbeit von Frank et al. und wurde erstmals für New York City entwickelt [47]. Als Betrachtungsebenen dienen Stadtbezirksviertel. Damit ist eine relativ detaillierte Betrachtung möglich und die Ergebnisse sind an andere bereits vorliegende Studien anschließbar. Der berechnete Index hat niedrige Werte bei einer angenommenen niedrigen Walkability und hohe Werte bei einer fußgängerfreundlicheren Umgebung. Als Beispiel soll hier das Ergebnis der Walkability in München gezeigt werden, in dem Grünflächen und unbewohnte Flächen maskiert wurden (Abb. 5).

Kartendarstellungen wie auch die Berechnung des Index an sich bieten ein Instrument zur integrierten Betrachtung der Verhaltensumwelt der Bürgerinnen und Bürger im urbanen Raum anhand klar strukturierter Kriterien in einem umfassenden methodischen Rahmen. Das Konzept eröffnet umfangreiche Möglichkeiten zur Weiterentwicklung des bestehenden Index. Hierzu bietet sich ein interdisziplinärer Ansatz an, um zielgruppenspezifische Belange sowohl aus der Perspektive der Gesundheitsvorsorge als auch der Umweltvorsorge möglichst vollständig zu erfassen. Speziell für München soll der Index weiterentwickelt werden unter Implementierung von zielgruppenspezifischen Elementen (Kinder, ältere Menschen, vulnerable Gruppen), unter Einbezug von OpenStreetMap-Daten und Haltestellen des öffentlichen Personennahverkehrs, und mit Analysemethoden wie dem Kernel Density Estimator zur leichteren Darstellung der einzelnen Variablen im Index.

## Diskussion: Quo vadis Geografie in der GBE

In den 6 Beispielen der verschiedenen GBE-Ebenen wurde gezeigt, dass geografische Methoden zu unterschiedlichen Themengebieten mit diversen Methoden eingesetzt werden können. Selbst einfachere Kartendarstellungen, wie im Gesundheitsatlas Baden-Württemberg oder bei der Analyse der Schuleingangsuntersuchung in München, können informieren und Bereiche identifizieren, in denen weitere Projekte sinnvoll wären.

Allerdings ist technisch gesehen sehr viel mehr möglich, von Disease Mapping über Erreichbarkeitsanalysen über Netzwerkanalysen oder räumliche Statistik. Der Walkability-Index für München, aber auch die Influenza-Surveillance (mit Glättungsverfahren und temporären Verläufen) zeigen hier Ansätze. Dass ähnliche Methoden nicht viel häufiger angewandt werden, kann vornehmlich mit der eingeschränkten Datenverfügbarkeit begründet werden.

Primärstudien haben oft nicht die Fallzahlgröße, um kleinräumige Analysen zuzulassen. Auch ist die Problematik des Datenschutzes zu beachten. Eine gute Quelle wären die Daten der gesetzlichen Krankenkassen oder die der kassenärztlichen Vereinigungen, um höhere Fallzahlen analysieren zu können und auch große Teile der Bevölkerung abbilden zu können. Auch hier sind der Datenschutz und der Datenzugang oft eine starke Limitation, da diese nicht kleinsträumig, d. h. unter der Ebene der Kreise und kreisfreien Städte, zur Verfügung gestellt werden können.

Im kommunalen Bereich kommt noch dazu, dass es auf Ebene unterhalb der Stadtgrenzen entweder keine Daten gibt oder aber diese in unterschiedlichen geografischen Einheiten erhoben werden (bspw. Postleitzahlen oder Stadtbezirke), die nicht miteinander kompatibel sind. Ein weiteres Problem wird an den Stadträndern einer Großstadt bzw. generell an Grenzen administrativer Einheiten sichtbar. Aus Sicht von Bürgerinnen und Bürgern spielen administrative Grenzen, wie etwa die Stadtgrenze im Hinblick auf die Nutzung gesundheitsbezogener Einrichtungen wie etwa Arztpraxen, Physiotherapie etc., im Alltag keine Rolle. Dennoch werden Planungen auf kommunaler Ebene in der Regel auf der Basis von Daten durchgeführt, die nicht über diese Stadtgrenzen hinausgehen. Methodisch werden diese beiden Problematiken (unterschiedliche Grenzziehungen innerhalb eines Gebietes, die nicht deckungsgleich sind, und die Größe der Einheiten an sich) unter dem Begriff des Modifiable Areal Unit Problem (MAUP) zusammengefasst [48]. Diese Problematiken könnten durch die Schaffung gesundheitsrelevanter Planungseinheiten überwunden werden oder durch erweiterte Methoden (wie geografische Glättungsverfahren) abgeschwächt werden.

Wenn diese Hindernisse überwunden werden könnten, würde die bestehende GBE davon profitieren und es könnten weitere Bereiche einbezogen werden, in denen die geografische Public-Health- und Versorgungsforschung die GBE ergänzen könnte.

Insbesondere im Bereich der ärztlichen oder therapeutischen Versorgung wären Erreichbarkeitsanalysen wichtig, um Informationen über Versorgungsdisparitäten sowohl kommunal als auch überregional (bis hin zu landesweiten Analysen) zu gewinnen und dementsprechend Ansatzpunkte für notwendige oder sinnvolle Maßnahmen zu schaffen. Hier können auch zielgruppenspezifische Ansätze durchgeführt werden, um gezielte neue Programme zu entwickeln. Um das Thema der Erreichbarkeitsanalysen methodisch und inhaltlich auf hohem Niveau durchführbar zu machen, ist eine *Gute Praxis Erreichbarkeitsanalysen* bereits in der Planung [49].

Zusammenfassend lässt sich festhalten, dass die GBE stark von gesundheitsgeografischen Methoden und Konzepten profitieren kann, nicht zuletzt, um die Gesundheit und/oder die Versorgung der Bevölkerung zu verbessern. Die Berücksichtigung des Raumes in Form verschiedener regionaler Betrachtungsebenen generiert einen hohen und komplementären Erkenntnisgewinn und dient der gezielteren Umsetzung gesundheitspolitischer Maßnahmen. Datenschutzrichtlinien limitieren jedoch das Potenzial einer detaillierteren räumlichen Betrachtung von Gesundheit bzw. einer Gesundheitsberichterstattung. Diesbezüglich kann der Wunsch geäußert werden, ein gesundes Maß an Datenschutz beizubehalten, jedoch nicht darüber hinauszugehen. Auch ein vereinfachter Zugang zu kleinräumigen Daten und die Berücksichtigung geografischer Kenngrößen in Studiendesigns können das Potenzial dieser Betrachtungs- und Analysemöglichkeiten stark unterstützen.
